# Large-scale identification of lysine acetylated proteins in vegetative hyphae of the rice blast fungus

**DOI:** 10.1038/s41598-017-15655-4

**Published:** 2017-11-10

**Authors:** Xiaomei Sun, Zhigang Li, Hang Liu, Jun Yang, Wenxing Liang, You-Liang Peng, Jinguang Huang

**Affiliations:** 1 College of Animation and Communication, Qingdao Agricultural University, Qingdao, 266109 China; 2 State Key Laboratory of Agrobiotechnology, and Ministry of Agriculture Key Laboratory of Pest Monitoring and Green Management, College of Plant Protection, China Agricultural University, Beijing, 100193 China; 3 The Key Laboratory of Integrated Crop Pest Management of Shandong Province, College of Plant Health and Medicine, Qingdao Agricultural University, Qingdao, 266109 China

## Abstract

Lysine acetylation is a major post-translational modification that plays important regulatory roles in diverse biological processes to perform various cellular functions in both eukaryotes and prokaryotes. However, roles of lysine acetylation in plant fungal pathogens were less studied. Here, we provided the first lysine acetylome of vegetative hyphae of the rice blast fungus *Magnaporthe oryzae* through a combination of highly sensitive immune-affinity purification and high-resolution LC-MS/MS. This lysine acetylome had 2,720 acetylation sites in 1,269 proteins. The lysine acetylated proteins were involved indiverse cellular functions, and located in 820 nodes and 7,709 edges among the protein-protein interaction network. Several amino acid residues nearby the lysine acetylation sites were conserved, including K^ac^R, K^ac^K, and K^ac^H. Importantly, dozens of lysine acetylated proteins are found to be important to vegetative hyphal growth and fungal pathogenicity. Taken together, our results provided the first comprehensive view of lysine acetylome of *M.oryzae* and suggested protein lysine acetylation played important roles to fungal development and pathogenicity.

## Introduction

Protein acetylation is an evolutionarily conserved post-translational modification in both eukaryotes and prokaryotes^1^. Acetylation of lysine residues in proteins is a dynamic and reversible process that was first discovered in histone proteins nearly fifty years ago^2^.Acetylation of histones and other transcription factors in nucleus has been extensively studied in regulation of gene transcription^3^. After discovery of lysine acetylation in non-histone proteins, it is believed that the extent of this modification is not restricted to nuclei, which has greatly expanded our understanding on functions of this modification^4,5^.In recent ten years, lysine acetylation has been found to occur in almost every compartment of a cell, such as the mitochondria and the cytoplasm^6–8^, and to play important roles in various cellular processes including protein-protein interactions^9^, enzymatic activity^10,11^, metabolic pathways^9,10,12–14^, cell morphology^7^, calorie restriction^15,16^, and protein-nucleic acid interactions^17,18^. Therefore, lysine acetylation is believed to be a main signaling modulator due to its widely occurrences and diverse cellular functions^19^.

Proteome-wide lysine acetylation profiles in many prokaryotes^2,12,20–27^ and eukaryotes^6–8,15,28–34^ have been investigated by mass spectrometry (MS)-based proteomics. These studies have provided definite evidences about biological functions of lysine acetylation. Identification of acetylome on proteomic level greatly increases the knowledge of lysine acetylated proteins and expands global view of their functional landscape^28^. However, few studies have been reported about the lysine acetylome in plant fungal pathogens. Until now, only two recent papers reported proteome-wide analysis of lysine acetylation in plant pathogens *Botrytis cinerea* and *Fusarium graminearum* ^28,35^.

In this study, we performed large-scale identification of lysine acetylated proteins in the rice blast fungus, *Magnaporthe oryzae*, which can cause rice blast, one of the most devastating diseases on rice throughout the world^36–38^. We identified 2,720 lysine acetylation sites in 1,269 proteins, which was account for about 10.3% of the total proteins in this fungus. Several amino acid residues surrounding the lysine acetylation sites were conserved, including K^ac^R, K^ac^K, and K^ac^H. The lysine acetylated proteins were predicted to involve in diverse cellular functions and located in 820nodes and 7,709 edges among the protein-protein interaction network. Dozens of lysine acetylated proteins are found to be important to vegetative hyphal growth and fungal pathogenicity. In summary, our results provided the first lysine acetylome of *M. oryzae* and suggested protein lysine acetylation played important roles to fungal development and pathogenicity.

## Results

### Proteome-wide features of lysine acetylation sites and proteins

**Figure 1:**
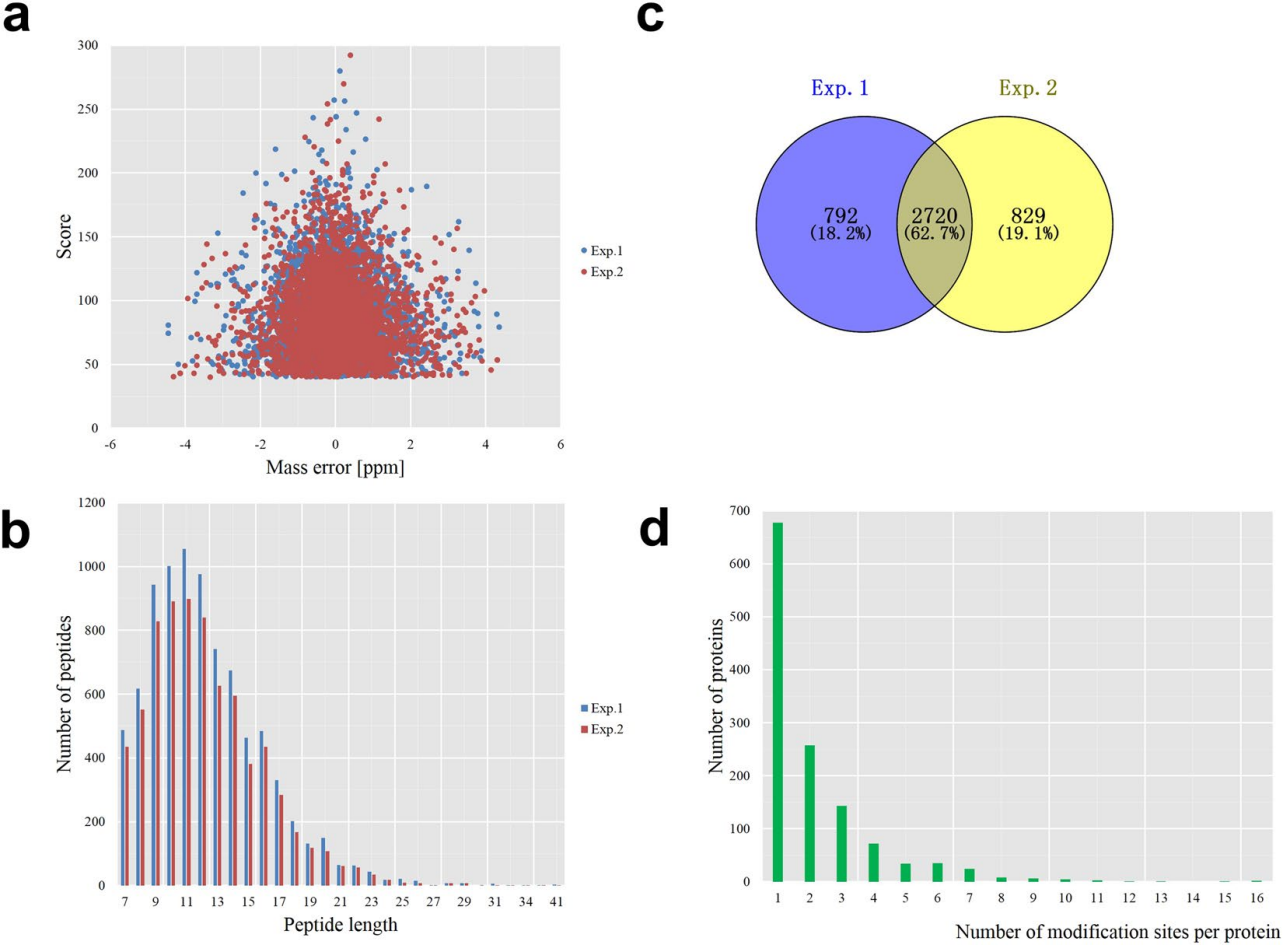
QC validation of MS data and summary of acetylated proteins. (**a**) Mass error distribution of all identified peptides, (**b**) Peptide length distribution. (**c**) Venn diagram representing the number of acetylation sites for the two biological replicate analysis, (**d**) Distribution of acetylated proteins based on their number of acetylation sites.

To perform large scale lysine acetylome analysis of *M. oryzae*, an integrated approach including protein extraction, trypsin digestion, HPLC fractionation, affinity enrichment, and high-resolution LC-MS/MS with following database search and bioinformatics analysis was employed in this study. Samples were originated from vegetative hyphae shaken in liquid complete medium of strain P131^39^. Two biological replicates were performed to confirm the integrity of our data. When the MS data was obtained, we searched them against the *M. oryzae* database concatenated with reverse decoy database, and performed QC validation. Firstly, we checked the mass error of all the identified peptides. The distribution of mass error was near zero and most of them were less than 0.02 Da, which mean mass accuracy of the MS data fitted the requirement (Fig. 1a). Secondly, length of most peptides distributed between 8 and 20, which agreed with the property of tryptic peptides (Fig. 1b). Therefore, the sample preparation reached the standard.

Acetylation sites detected by both of two replicates were used in our analysis (Fig. 1c). A total of 2,720 lysine acetylation sites were finally identified, and these lysine acetylation sites were distributed in 1,269proteins (Fig. 1d and Table S1), which was account for about 10.3% of the total predicted proteins in *M. oryzae* ^39,40^. The number of lysine acetylation sites in proteins was diverse and distributed from one to ten (Fig. 1d). There were 678 proteins with only one acetylation site, which was account for 53.4% of acetylated proteins. Meanwhile, 257 (20.3%), 143 (11.2%), 72 (5.7%), and 34 (2.7%) proteins contained two, three, four, and five acetylated sites respectively (Fig. 1d). Other 6.7% of modified proteins contain more than five acetylated sites.

### Lysine acetylation motif investigation

**Figure 2:**
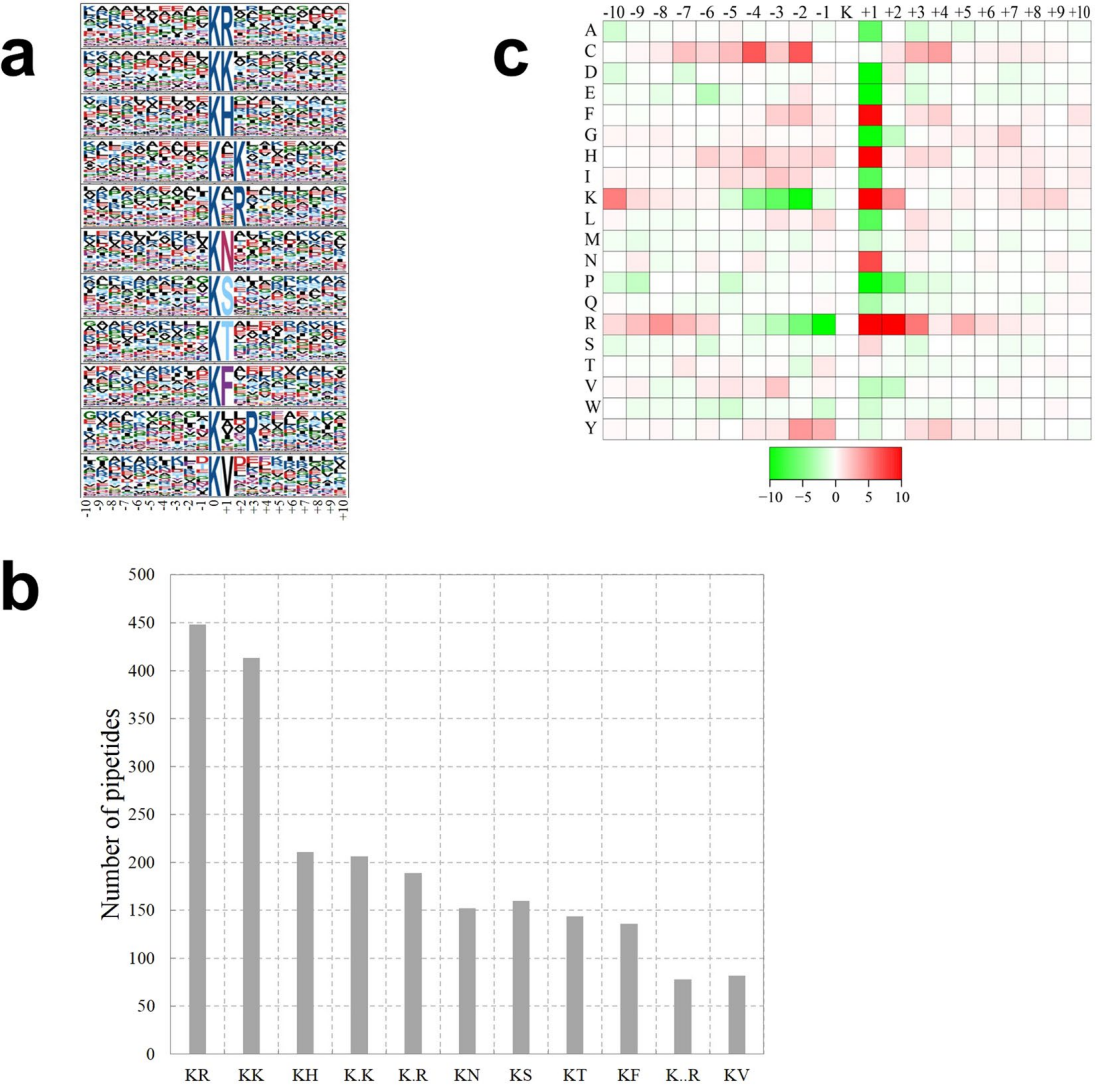
Acetylation motifs and conservation of acetylation sites. (**a**) Acetylation motifs and conservation of acetylation sites. (**b**) Number of identified modification sites in each acetylated protein. (**c**) Heat map of the amino acid compositions of the acetylation sites.

To determine the pattern of lysine acetylation sites, software motif-x was used to analyze amino acid sequences from the −10 to + 10 positions of the identified acetylation sites. As shown in Fig. 2a, a total of 11conserved motifs were identified in the acetylated proteins, i.e. K^ac^R, K^ac^K, K^ac^H, K^ac^XK, K^ac^XR, K^ac^N, K^ac^S, K^ac^T, K^ac^F, K^ac^XXR, K^ac^V(K^ac^ represented the acetylated lysine and X represented a random amino acid residue).Among them, the motifs K^ac^R, K^ac^K, and K^ac^H were highly conserved and ranked at the top three, which were count for 39.4% of the all identified acetylated peptides (Fig. 2b).Most of these motifs were conserved among other species^2,7,14,15,23–27,30,32,34^.In these motifs, several amino acid residues were conserved, for instance, Arginine(R), Lysine (K), Histidine (H), and Asparagine(N) were located downstream of acetylated lysines. The heat map of amino acid compositions surrounding the acetylation sites were generated (Fig. 2c).While the enrichment of amino acid residues R, H, and K were observed in the + 1 position, and amino acid residue Cysteine(C)was observed in the −1 position.

### Annotation of acetylated proteins

**Figure 3:**
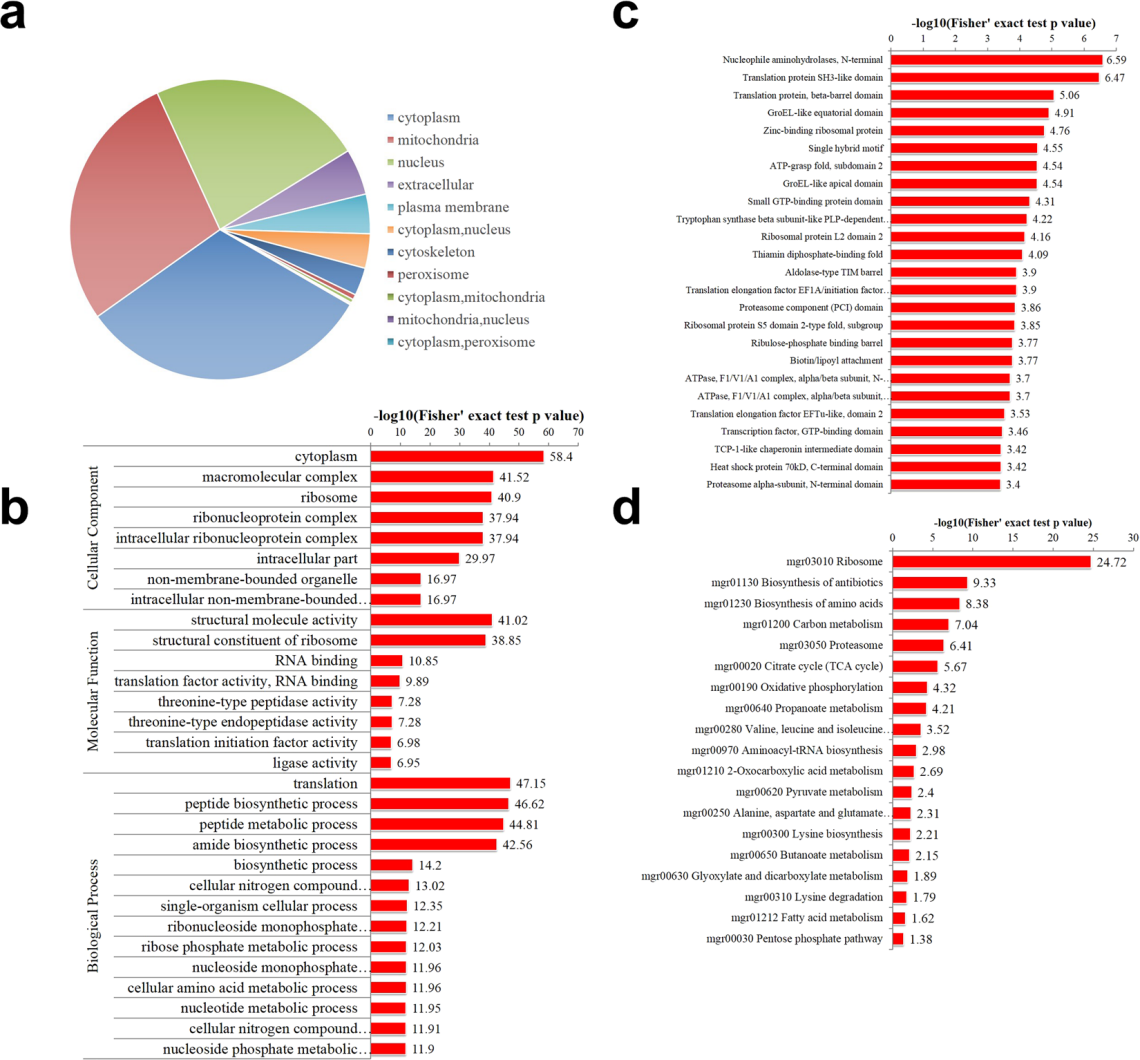
Characteristics of identified acetylated proteins. (**a**) Pie chart showing the protein subcellular localization of acetylated proteins. (**b**) Gene Ontology functional classifications of acetylated proteins, which were based on molecular function, cellular component and biological process. (**c**) Protein domain enrichment analysis of acetylated protein. (**d**) KEGG pathway-based enrichment analysis of acetylated proteins.

To investigate functions of the lysine acetylated proteins, we first annotated their subcellular localization with the WoLFPSORT. As shown in Fig. 3a, most of the lysine acetylated proteins were located in the cytoplasm (31.8%), the mitochondria (30.0%), and the nucleus (23.1%).For the rest lysine acetylated proteins, 63 ones (5.0%) and 54 ones (4.3%) were located in the extracellular space and the plasma membrane, respectively. These results suggested diverse subcellular localization of lysine acetylated proteins occurred, especially the intracellular compartments.

We then classified the lysine acetylated proteins based on their predicted functions. All proteins were first annotated by GO terms. As shown in Fig. 3b, the most significantly enriched biological process was translation (116 proteins). The main molecular function was structural molecule activity (89 proteins).We also investigated protein domains and found that functional domains related to nucleophile amino hydrolases and translation protein SH3-like domain were significantly enriched in the lysine acetylated proteins (Fig. 3c). KEGG pathway analyses showed that 20 pathways were enriched for acetylated proteins (Fig. 3d). The most abundant one was the ribosome pathway, which contains 83acetylated proteins. Other enriched pathways included biosynthesiss, carbon metabolism, and so on.

### Protein-protein interaction network feature

**Figure 4:**
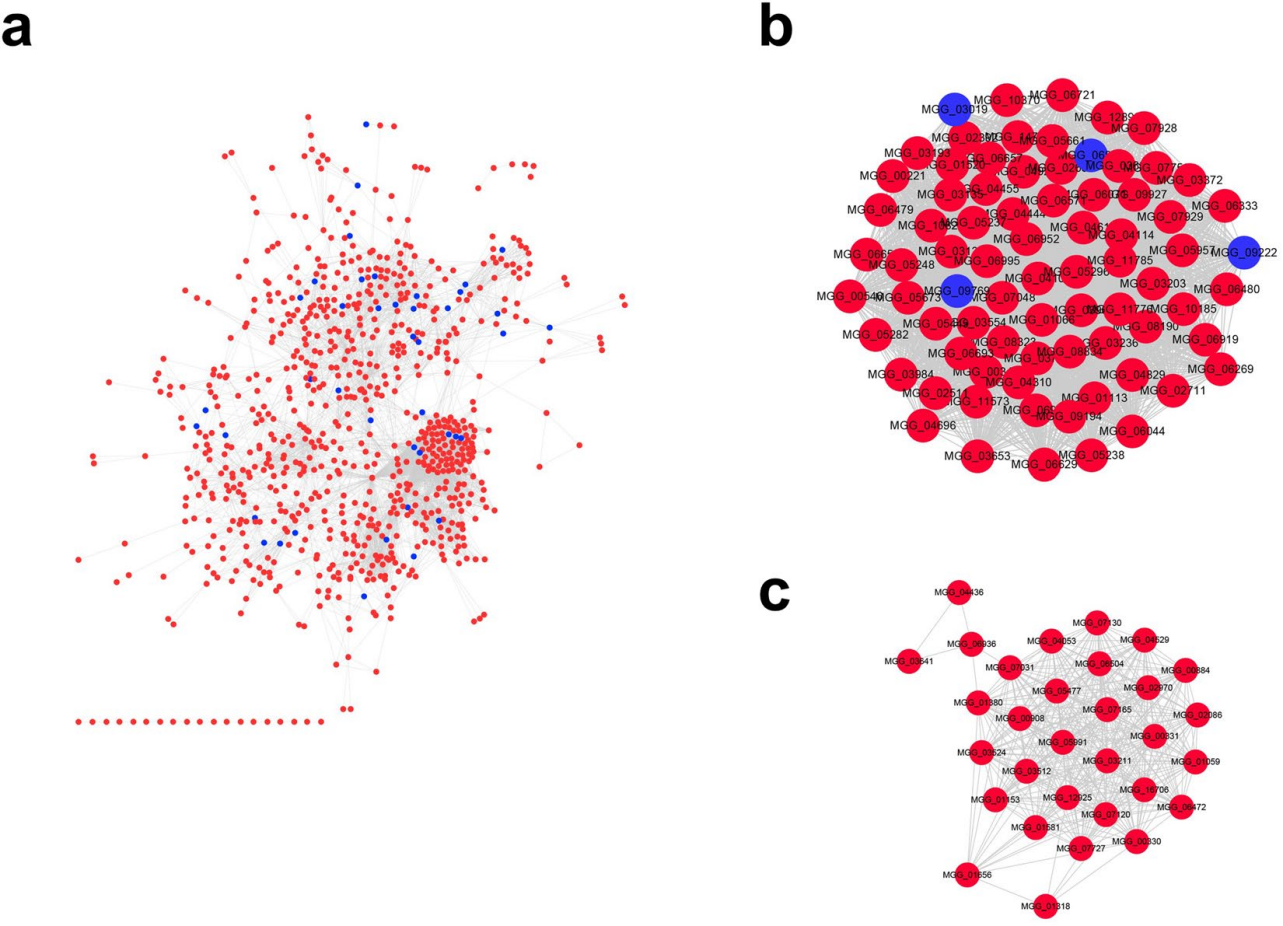
Protein-protein interaction networks of identified acetylated proteins. The red and blue nodes in networks indicated acetylated proteins and non-acetylation proteins. (**a**) The overview of interaction network of acetylated proteins. (**b**) and (**c**) indicated interaction network of acetylated proteins associated with ribosome and proteasome, respectively.

To further understand biological process regulated by acetylation, protein-protein interaction (PPI) network analyses on lysine acetylated proteins were performed. PPI network including all protein interactions in different developmental stages was established with the search tool for the retrieval of interacting genes and/or proteins (STRING) database (Fig. 4a; Table S2). We found that the lysine acetylated proteins formed a highly organized network of interacting proteins. We performed network analyses on the established network at high STRING confidences. Overall, the interacting network of the lysine acetylated proteins had significantly more interactions than expected, and this PPI network contained 820 nodes with 7,709 edges, in which the average node degree was 18.8.

The established PPI network was then analyzed by MCODE^41^, and38highly inter-connected clusters within the network were identified (Table S2). Several dominant clusters involved in lots of functionally related proteins. The most top cluster was ribosome. Among the 77 ribosome proteins, 73 proteins were lysine acetylated (Fig. 4b; Table S2). The second top was proteasome. Among the 31 proteasome proteins, 30 proteins were lysine acetylated (Fig. 4c; Table S2). In addition to these top clusters, the largest group of clusters was related to metabolic processes, e.g. cluster_4, cluster_5, cluster_6, cluster_10, cluster_11, and so on. Moreover, several identified inter-connected clusters were related with protein processing. For instance, cluster_7 was related with protein folding, cluster_8 and cluster_26were related with translation initiation, cluster_30 and cluster_37 were related with protein transport. Some clusters contained proteins with other functions. For example, cluster_12 was involved in MAPK signaling pathway, cluster_13 was composed by serine/threonine-protein phosphatases.

### Dozens of lysine acetylated proteins are involved in vegetative hyphal growth and pathogenicity

To investigate roles of the identified 1,269 lysine acetylated proteins in fungal growth and pathogenicity, we retrieved these proteins to gene repositories built from the literature annotations reported throughout the last two decades. Thirty-five reported proteins were found to be important to vegetative hyphal growth (Table 1). Importantly, 27ones were essential to pathogenicity or important for virulence. Pfam and KEGG analysis showed that some lysine acetylated proteins were involved in signaling transduction pathway. For examples, Chm1 encodes a PKA protein kinase and MoCmk1 encodes a protein kinase, both of which were important to fungal growth and pathogenicity^42^.Some lysine acetylated proteins were involved in amino acid metabolism, for examples, 5-methyltetrahydropteroyl triglutamate-homocysteine S-methyltransferase MoMet6 and L-aminoadipatesemialdehyde dehydrogenase MoLys2, and they were also important to fungal growth and plant infection^43,44^. To test whether other acetylated proteins identified in this study were involved in vegetative hyphal growth, we searched our proteins against an ATMT mutant library reported previously^45^.We found that 16 disrupted genes were potential to be important for vegetative hyphal growth, some of which were also contribute to fungal pathogenicity (Table S3). Taken together, these findings suggested that dozens of lysine acetylated proteins were involved into vegetative hyphal growth, and several of them were involved in fungal pathogenicity.

**Table 1:** List of acetylated proteins involved in vegetative hyphal growth and fungal pathogenicity.

Gene ID	Gene Name	Annotation	Vegetative hyphal growth	Fungal pathogenicity Reference
MGG_13013	MoChs6	chitin synthase	slow	loss of pathogenicity. Osmond *et al*.^54^
MGG_06712	MoMet6	cobalamine-independent methionine synthase	slow	loss of pathogenicity. Saint-Macary *et al*.^43^
MGG_12868	Ech1	enoyl-CoA hydratase	slow	loss of pathogenicity. Patkar *et al*.^55^
MGG_05201	Mgb1	guanine nucleotide-binding protein beta subunit	slow	loss of pathogenicity. Li *et al*.^56^
MGG_04943	Mps1	MAPK protein kinase	slow	loss of pathogenicity. Qi *et al*.^57^
MGG_06320	Chm1	PAKA protein kinase	slow	loss of pathogenicity. Li *et al*.^47^
MGG_08144	MoYpt7	Rab small GTPase	slow	loss of pathogenicity. Liu *et al*.^58^
MGG_06868	MoIlv2	acetolactate synthase	slow	reduced virulence. Du *et al*.^59^
MGG_01722	MoCap1	adenylyl cyclase-associated protein	slow	reduced virulence. Zhou *et al*.^60^
MGG_09912	MoCmk1	calcium/calmodulin-dependent protein kinase	slow	reduced virulence. Takano *et al*.^61^
MGG_06361	MoDnm1	dynamin-related protein	slow	reduced virulence. Zhong *et al*.^62^
MGG_04978	MoSkp1	E3 ubiquitin ligase	slow	reduced virulence. Prakash *et al*.^63^
MGG_01744	ETFB	electron-transferring flavoprotein	slow	reduced virulence. Li *et al*.^64^
MGG_01719	ETFDH	electron-transferring flavoprotein dehydrogenase	slow	reduced virulence. Li *et al*.^64^
MGG_00365	MagB	guanine nucleotide-binding protein alpha subunit	slow	reduced virulence. Fang *et al*.^65^
MGG_01661	Mokmt3	histone lysine methyltransferase	slow	reduced virulence. Pham *et al*.^66^
MGG_02611	MoLys2	L-aminoadipatesemialdehyde dehydrogenase	slow	reduced virulence. Chen *et al*.^67^
MGG_08203	MoMbf1	multiprotein bridging factor	slow	reduced virulence. Fan *et al*.^68^
MGG_04489	Mnh6	non-histone chromosomal protein	slow	reduced virulence. Lu *et al*.^69^
MGG_07190	MoPmt2	o-mannosyltransferase	slow	reduced virulence. Guo *et al*.^70^
MGG_07135	MoPyr5	orotate phosphoribosyl transferase	slow	reduced virulence. Qi *et al*.^71^
MGG_04545	MoCcp1	peroxidase	slow	reduced virulence. Du *et al*.^72^
MGG_05089	MoVps35	retromer	slow	reduced virulence. Zheng *et al*.^73^
MGG_06135	MoSec. 4	small GTPase	slow	reduced virulence. Zheng *et al*.^74^
MGG_05133	MoCrz1	transcriptional regulator	slow	reduced virulence. Zhang *et al*.^75^
MGG_08556	MoVelA	velvet factor	slow	reduced virulence. Wickramage.^76^
MGG_08623	MoGls2	neutral alpha-glucosidase	slow	reduced virulence. Li *et al*.^77^
MGG_09263	MoCod2	C6 zinc finger domain-containing protein	slow	wild type. Chung *et al*.^78^
MGG_01620	MoVelB	developmental regulator	slow	wild type. Kim *et al*.^79^
MGG_08370	MoGel3	glucan elongation factor	slow	wild type. Samalova *et al*.^80^
MGG_11861	MoGel4	glucan elongation factor	slow	wild type. Samalova *et al*.^80^
MGG_12822	Pgi1	glucose-6-phosphate isomerase	slow	wild type. Pan *et al*.^81^
MGG_02755	Nut1	nitrogen regulatory protein	slow	wild type. Froeliger *et al*.^82^
MGG_06148	Mfp1	peroxisomal hydratase dehydrogenase epimerase	slow	wild type. Wang *et al*.^83^
MGG_09531	MoRga4	Rho-GTPase-activating protein	slow	wild type. Terauchi *et al*.^84^

## Discussion

Researchers have remarked that lysine acetylation can provide a new target for the development of effective drugs or vaccines based on an understanding of its regulatory mechanism^46^. However, studies summarized its function in plant pathogen fungi were almost entirely absent. As apioneering research of lysine acetylome in the rice blast fungus, this study helped researchers to understand the importance of acetylated processes in plant pathogen fungi, and provided useful and accessible data for further study in the biological field. This lysine acetylome contains 2,720 acetylation sites in 1,269proteins, which occupied about 10.3% of the total predicted proteins in this fungus. As compared with the thousands acetylated proteins discovered by *in silico* analysis techniques, we speculated that many lysine sites were dynamic acetylated during different development stages and under diverse environmental stimulus. Therefore, we will continue to gather acetylation data to obtain global view of the lysine acetylome in *M. oryzae*, and disseminated them to researchers. Moreover, our ability to detect acetylated proteins in different conditions and development stages is giving the biological researchers unprecedented access to understand infection-related morphogenesis or asexual development. Together, these findings widen roles of reversible acetylation in *M. oryzae* and open up new possibilities for investigations in the field.

By comparing characterizes of the lysine acetylated proteins, such as motifs, subcellular localizations, annotated functions, among different species, we found most of them are highly conserved. Moreover, all of the annotated protein-protein interaction networks among the lysine acetylated proteins have been reported in other species^9–18,28,35^. So, these analyses suggested conservation of protein lysine acetylation during evolution. However, 173 proteins with lysine acetylated sites were annotated as function unknown (Table S1). Dissection roles of these new proteins might enrich overview of the lysine acetylome of *M. oryzae*.

We found dozens of previously reported proteins important to vegetative hyphal growth were lysine acetylated. These proteins were involved in diverse functions, such as signal transduction, amino acid metabolism, energy transfer, cytoskeleton, transcription regulation, and so on. Importantly, two protein kinases, MoCmk1and Chm1, were reported to play important roles in fungal pathogenicity^47–49^. Unfortunately, roles of the acetylated lysine sites have not been functionally investigated. Recently, dynamic crosstalk between receptor tyrosine kinases and lysine acetylation were revealed by quantitative profiling of lysine acetylation in cultured carcinoma cell lines^50^. Moreover, phosphorylation and lysine acetylation cross-talk in a kinase motif associated with myocardial ischemia and cardioprotection were dissected by structure-based analysis^51^. Therefore, the acetylated lysine sites identified in our study will provide valuable information to investigate the interactions between lysine acetylation and other post-translation modifications. Furthermore, over 10novel proteins with predicted protein kinase domains were detected to contain lysine acetylated sites in this study. It will be interesting to characterize their functions on developments and plant infection and roles of lysine acetylated sites. Recently, a circadian-regulated protein Twilight/Twl, which plays key roles in conidiation and pathogenesis in *M. oryzae*, was found to contain one lysine acetylated site^52^. The de-acetylated form of Twilight/Twldriven by light-induced phosphorylation leads to its translocation from cytoplasm into nucleus. Because the acetylated Twilight/Twl was only detected in dark condition, it seems reasonable that the lysine acetylated site in Twilight/Twl could not be identified in our study. This study also strongly suggested important roles of lysines acetylation during development and pathogenicity.

Taken together, our study provides a comprehensive view of lysine acetylated sites during vegetative hyphal growth in *M. oryzae*. It will be helpful to understand roles of the protein with acetylated lysine at the post-translational modification level.

## Materials and Methods

### Protein extraction

Samplewas first grinded by liquid nitrogen, then the cell powder was transferred to 5 ml centrifuge tube and sonicated three times on ice using a high intensity ultrasonic processor (Scientz) in lysis buffer (8 M urea, 1% Triton-100, 65 mM DTT, and 0.1% Protease Inhibitor Cocktail). The remaining debris was removed by centrifugation at 20,000 g at 4 °C for 10 min. Finally, the protein was precipitated with cold 15% trifluoroacetic acid (TFA) for 2 h at −20 °C. After centrifugation at 4 °C for 10 min, the supernatant was discarded. The remaining precipitate was washed with cold acetone for three times. The protein was re-dissolved in buffer (8 M urea, 100 mM NH_4_CO_3_, pH 8.0) and the protein concentration was determined with 2-D Quant kit (GE Healthcare) according to the manufacturer’s instructions.

### Trypsin digestion

For digestion, the protein solution was reduced with 10 mM DTT for 1 h at 37 °C and alkylated with 20 mM iodoacetamide (IAA) for 45 min at room temperature in darkness. For trypsin digestion, the protein sample was diluted by adding 100 mM NH_4_CO_3_ to urea concentration less than 2 M. Finally, trypsin (Promega) was added at 1:50 trypsin-to-protein mass ratio for the first digestion overnight and 1:100 trypsin-to-protein mass ratio for a second 4 h-digestion.

### HPLC Fractionation

The sample was then fractionated into fractions by high pH reverse-phase HPLC using Agilent 300Extend C18 column (5 μm particles, 4.6 mm ID, 250 mm length). Briefly, peptides were first separated with a gradient of 2% to 60% acetonitrile in 10 mM ammonium bicarbonate pH 10 over 80 min into 80 fractions. The peptides were then combined into 8 fractions and dried by vacuum centrifuging.

### Affinity Enrichment

To enrich acetylated lysine (Kac) peptides, tryptic peptides dissolved in NETN buffer (100 mMNaCl, 1 mM EDTA, 50 mMTris-HCl, 0.5% NP-40, pH 8.0) were incubated with pre-washed antibody beads (PTM Biolabs) at 4 °C overnight with gentle shaking. The beads were washed four times with NETN buffer and twice with ddH_2_O. The bound peptides were eluted from the beads with 0.1% TFA. The eluted fractions were combined and vacuum-dried. The resulting peptides were cleaned with C18 ZipTips (Millipore) according to the manufacturer’s instructions, followed by LC-MS/MS analysis.

### LC-MS/MS Analysis

Three parallel analyses for each fraction were performed. Peptides were dissolved in 0.1% formic acid (FA), directly loaded onto a reversed-phase pre-column (Acclaim PepMap 100, Thermo Scientific). Peptide separation was performed using a reversed-phase analytical column (Acclaim PepMap RSLC, Thermo Scientific). The gradient was comprised of an increase from 6% to 23% solvent B (0.1% FA in 98% acetonitrile) for 24 min, 23% to 35% for 8 min and climbing to 80% in 4 min then holding at 80% for the last 4 min, all at a constant flow rate of 280 nl/min on an EASY-nLC 1000 UPLC system, the resulting peptides were analyzed by Q Exactive^TM^ Plus hybrid Quadrupole-Orbitrap mass spectrometer (ThermoFisher Scientific).

### Database Search

The resulting MS/MS data was processed using MaxQuant with integrated Andromeda search engine (v.1.4.1.2). Tandem mass spectra were searched against *Magnaporthe oryzae* (14,835 sequences) database concatenated with reverse decoy database. Trypsin/P was specified as cleavage enzyme allowing up to 3 missing cleavages, 4 modifications per peptide and 5 charges. Mass error was set to 10 ppm for precursor ions and 0.02 Da for fragment ions. Carbamidomethylation on Cys was specified as fixed modification and oxidation on Met, acetylation on Lysine and acetylation on protein N-terminal were specified as variable modifications. False discovery rate (FDR) thresholds for protein, peptide and modification site were specified at 1%. Minimum peptide length was set at 7. All the other parameters in MaxQuant were set to default values. The site localization probability was set as >0.75.

### Annotation and functional enrichment analysis

Gene Ontology (GO) annotation was derived from the UniProt-GOA database (http://www.ebi.ac.uk/GOA/). Proteins were classified by GO annotation into three categories: biological process, cellular compartment and molecular function. Identified proteins domain functional descriptions were annotated by InterPro domain database (http://www.ebi.ac.uk/interpro/). KEGG database (http://www.genome.jp/kegg/) was used to identify enriched pathways. These pathways were classified into hierarchical categories according to the KEGG website. A two-tailed Fisher’s exact test was employed to test the enrichment of the identified acetylated protein against all database proteins. Correction for multiple hypothesis testing was carried out using standard FDR methods. The GO, domains, and pathways with a corrected *p*-value < 0.05 are considered significant. WoLFPSORT (http://www.genscript.com/wolf-psort.html) was used to predict subcellular localization.

### Motif analysis

Motif analysis was performed with softwaremotif-x^53^ by analyzing the model of sequences constituted with amino acids in specific positions of modifier-21-mers (10 amino acids upstream and downstream of the site) in all protein sequences. All of the protein sequences were used as background database parameter, and other parameters with default.

## Electronic supplementary material

Table S3

Table S1

Table S2

## References

[1] Weinert BT (2013). Lysine succinylation is a frequently occurring modification in prokaryotes and eukaryotes and extensively overlaps with acetylation. Cell Rep..

[2] Pan J (2014). Systematic analysis of the lysine acetylome in *Vibrio parahemolyticus*. J. Proteome Res..

[3] Kouzarides T (2007). Chromatin modifications and their function. Cell.

[4] Chen Y (2012). Quantitative acetylome analysis reveals the roles of SIRT1 in regulating diverse substrates and cellular pathways. Mol. Cell. Proteomics.

[5] Glozak MA (2005). Acetylation and deacetylation of non-histone proteins. Gene.

[6] Kim SC (2006). Substrate and functional diversity of lysine acetylation revealed by a proteomics survey. Mol. Cell.

[7] Choudhary C (2009). Lysine acetylation targets protein complexes and co-regulates major cellular functions. Science.

[8] Zhao S (2010). Regulation of cellular metabolism by protein lysine acetylation. Science.

[9] Hou J (2010). Phosphoproteome analysis of rat L6 myotubes using reversed-phase C18 prefractionation and titanium dioxide enrichment. J. Proteome Res..

[10] Nambi S (2013). Cyclic AMP-dependent protein lysine acylation in mycobacteria regulates fatty acid and propionate metabolism. J. Biol. Chem..

[11] Starai VJ (2004). Identification of the protein acetyltransferase (Pat) enzyme that acetylates acetyl-CoA synthetase in *Salmonella enterica*. J. Mol. Biol..

[12] Wang Q (2010). Acetylation of metabolic enzymes coordinates carbon source utilization and metabolic flux. Science.

[13] Guan KL (2011). Regulation of intermediary metabolism by protein acetylation. Trends Biochem. Sci..

[14] Xie LX (2015). Proteome-wide lysine acetylation profiling of the human pathogen *Mycobacterium tuberculosis*. Int. J. Biochem. Cell B..

[15] Henriksen P (2012). Proteome-wide analysis of lysine acetylation suggests its broad regulatory scope in *Saccharomyces cerevisiae*. Mol. Cell. Proteomics.

[16] Imai SI (2010). Ten years of NAD-dependent SIR2 family deacetylases: implications for metabolic diseases. Trends Pharmacol. Sci..

[17] Arif M (2010). Lysine acetylation: the tale of a modification from transcription regulation to metabolism. Chem biochem..

[18] Ren J (2016). Acetylation of Lysine 201 Inhibits the DNA-Binding Ability of PhoP to Regulate Salmonella Virulence. Plos Pathog..

[19] Norvell A (2010). Cell biology Rise of the Rival. Science.

[20] Liu L (2016). Acetylome analysis reveals the involvement of lysine acetylation in biosynthesis of antibiotics in *Bacillus amyloliquefaciens*. Sci. Rep..

[21] Ouidir T (2015). Proteomic profiling of lysine acetylation in *Pseudomonas aeruginosa* reveals the diversity of acetylated proteins. Proteomics.

[22] Liu F (2014). Acetylome analysis reveals diverse functions of lysine acetylation in *Mycobacterium tuberculosis*. Mol. Cell. Proteomics.

[23] Okanishi H (2013). Acetylome with structural mapping reveals the significance of lysine acetylation in *Thermusthermophilus*. J. Proteome Res..

[24] Zhang K (2013). Comprehensive profiling of protein lysine acetylation in *Escherichia coli*. J. Proteome Res..

[25] Kim D (2013). The acetylproteome of Gram-positive model *bacterium Bacillus* subtilis. Proteomics.

[26] Wu X (2013). Differential lysine acetylation profiles of *Erwiniaamylovora* strains revealed by proteomics. J. Proteomics.

[27] Lee DW (2013). Proteomic analysis of acetylation in thermophilic *Geobacilluskaustophilus*. Proteomics.

[28] Lv B (2016). Proteome-wide analysis of lysine acetylation in the plant pathogen *Botrytis cinerea*. Sci. Rep..

[29] Xiong Y (2016). A comprehensive catalog of the lysine-acetylation targets in rice (*Oryza sativa*) based on proteomic analyses. J. Proteomics.

[30] Nie Z (2015). Comprehensive profiling of lysine acetylation suggests the widespread function is regulated by protein acetylation in the silkworm. Bombyxmori. Proteomics.

[31] Nallamilli B (2014). Global analysis of lysine acetylation suggests the involvement of protein acetylation in diverse biological processes in rice (*Oryza sativa*). Plos One.

[32] Lundby A (2012). Proteomic analysis of lysine acetylation sites in rat tissues reveals organ specificity and subcellular patterns. Cell Rep..

[33] Finkemeier I (2011). Proteins of diverse function and subcellular location are lysine acetylated in Arabidopsis. Plant Physiol..

[34] Weinert BT (2011). Proteome-wide mapping of the Drosophila acetylome demonstrates a high degree of conservation of lysine acetylation. Sci. Signal.

[35] Zhou SY (2016). Systematic analysis of the lysine acetylomein*Fusarium graminearum*. BMC Genomics.

[36] Wilson RA (2009). Under pressure: investigating the biology of plant infection by *Magnaportheoryzae*. Nat. Rev. Microbiol..

[37] Talbot NJ (2003). On the trail of a cereal killer: Exploring the biology of *Magnaporthegrisea*. Ann. Rev. Microbiol..

[38] Dean R (2012). The Top 10 fungal pathogens in molecular plant pathology. Mol. plant pathol..

[39] Xue MF (2012). Comparative Analysis of the Genomes of Two Field Isolates of the Rice Blast Fungus *Magnaporthe oryzae*. Plos Genetics.

[40] Dean RA (2005). The genome sequence of the rice blast fungus *Magnaporthe grisea*. Nature.

[41] Bader GD (2003). An automated method for finding molecular complexes in large protein interaction networks. BMC Bioinformatics.

[42] Chen J (2008). Rac1 is required for pathogenicity and Chm1-dependent conidiogenesis in rice fungal pathogen *Magnaporthe grisea*. Plos Pathog..

[43] Saint-Macary ME (2015). Methionine biosynthesis is essential for infection in the rice blast fungus *Magnaporthe oryzae*. Plos One.

[44] Yan X (2013). The MET13 methylenetetrahydrofolate reductase gene is essential for infection-related morphogenesis in the rice blast fungus *Magnaporthe oryzae*. Plos One.

[45] Jeon J (2007). Genome-wide functional analysis of pathogenicity genes in the rice blast fungus. Nature Genetics.

[46] Ravindra KC (2009). Inhibition of lysine acetyltransferase KAT3B/p300 activity by anaturally occurring hydroxynaphthoquinone, plumbagin. J. Biol. Chem..

[47] Li L (2004). Two PAK kinase genes, CHM1 and MST20, have distinct functions in *Magnaporthe grisea*. Mol. Plant Microbe Interact.

[48] Zhao X (2005). A mitogen-activated protein kinase cascade regulating infection-related morphogenesis in *Magnaporthe grisea*. Plant Cell.

[49] Xu JR (1996). MAP kinase and cAMP signaling regulate infection structure formation and pathogenic growth in the rice blast fungus *Magnaporthe grisea*. Genes Dev..

[50] Bryson BD (2015). Quantitative Profiling of Lysine Acetylation Reveals Dynamic Crosstalk between Receptor Tyrosine Kinases and Lysine Acetylation. Plos One.

[51] Parker BL (2014). Structural basis for phosphorylation and lysine acetylation cross-talk in a kinase motif associated with myocardial ischemia and cardioprotection. J. Biol. Chem..

[52] Deng YZ (2015). Twilight, a Novel Circadian-Regulated Gene, Integrates Phototropism with Nutrient and Redox Homeostasis during Fungal Development. Plos Pathog..

[53] Chou MF (2011). Biological sequence motif discovery using motif-x. Curr. Protoc. Bioinformatics Chapter.

[54] Osmond BC (1999). Chitin synthase III: synthetic lethal mutants and “stress related” chitin synthesis that bypasses the CSD3/CHS6 localization pathway. Proceedings of the National Academy of Sciences.

[55] Patkar RN (2012). Mitochondrial β‐oxidation regulates organellar integrity and is necessary for conidial germination and invasive growth in *Magnaporthe oryzae*. Mol. Microbiol..

[56] Li Y (2010). MoRic8 is a novel component of G-protein signaling during plant infection by the rice blast fungus *Magnaporthe oryzae*. Mol. Plant Microbe Interact.

[57] Qi Z (2012). MoSwi6, an APSES family transcription factor, interacts with MoMps1 and is required for hyphal and conidial morphogenesis, appressorial function and pathogenicity of *Magnaporthe oryzae*. Mol. plant pathol..

[58] Liu XH (2015). The small GTPase MoYpt7 is required for membrane fusion in autophagy and pathogenicity of *Magnaporthe oryzae*. Environ. Microbiol..

[59] Du Y (2013). Acetolactate synthases MoIlv2 and MoIlv6 are required for infection‐related morphogenesis in *Magnaporthe oryzae*. Mol. plant pathol..

[60] Zhou X (2012). The Cyclase-associated protein Cap1 is important for proper regulation of infection-related morphogenesis in *Magnaporthe oryzae*. Plos Pathog.

[61] Takano Y (2000). The Colletotrichum lagenarium MAP kinase gene CMK1 regulates diverse aspects of fungal pathogenesis. Mol. Plant Microbe Interact.

[62] Zhong K (2016). MoDnm1 dynamin mediating peroxisomal and mitochondrial fission in complex with MoFis1 and MoMdv1 is important for development of functional appressorium in *Magnaporthe oryzae*. Plos Pathog.

[63] Prakash C (2016). Skp1, a component of E3 ubiquitin ligase, is necessary for growth, sporulation, development and pathogenicity in rice blast fungus (*Magnaporthe oryzae*). Mol. plant pathol..

[64] Li Y (2016). Functional characterization of electron-transferring flavoprotein and its dehydrogenase required for fungal development and plant infection by the rice blast fungus. Sci. Rep..

[65] Fang EG (2000). Site-directed mutagenesis of the magB gene affects growth and development in *Magnaporthe grisea*. Mol. Plant Microbe Interact.

[66] Pham KTM (2015). MoSET1 (Histone H3K4 Methyltransferase in *Magnaporthe oryzae*) regulates global gene expression during infection-related morphogenesis. PLoS Genetics.

[67] Chen Y (2014). MoLys2 is necessary for growth, conidiogenesis, lysine biosynthesis, and pathogenicity in *Magnaporthe oryzae*. Fungal Genet. Biol..

[68] Fan G (2017). Multiprotein-bridging factor 1 regulates vegetative growth, osmotic stress, and virulence in *Magnaporthe oryzae*. Curr. Genet..

[69] Lu JP (2007). Mnh6, a nonhistone protein, is required for fungal development and pathogenicity of *Magnaporthe grisea*. Fungal Genet. Biol..

[70] Guo, M. *et al*. The Pmt2p-mediated protein O-Mannosylation is required for morphogenesis, adhesive properties, cell wall integrity and full virulence of *Magnaporthe oryzae*. *Front. Microbiol*. 7 (2016). 10.3389/fmicb.2016.00630 PMC4852298 27199956

[71] Qi Z (2016). Orotate phosphoribosyl transferase MoPyr5 is involved in uridine 5′-phosphate synthesis and pathogenesis of *Magnaporthe oryzae*. Appl. Microbiol. Biot..

[72] Du X (2007). N-Acetyltransferase Mpr1 confers ethanol tolerance on Saccharomyces cerevisiae by reducing reactive oxygen species. Appl. Microbiol. Biot..

[73] Zheng W (2015). Retromer is essential for autophagy-dependent plant infection by the rice blast fungus. PLoS Genetics.

[74] Zheng, H. *et al*. The Small GTPase MoSec4 is involved in vegetative development and pathogenicity by regulating the extracellular protein secretion in *Magnaporthe oryzae*. *Front. Plant Sci*. 7 (2016). 10.3389/fpls.2016.01458 PMC5037964 27729922

[75] Zhang H (2009). MgCRZ1, a transcription factor of *Magnaporthe grisea*, controls growth, development and is involved in full virulence. FEMS Microbiol. Lett..

[76] Wickramage, A. S. Analysis of *Magnaporthe oryzae* homologs of *Histoplasma capsulatum* RYP genes. *The University of Arizona* (2013).

[77] Li M (2016). Glycoside Hydrolase MoGls2 Controls Asexual/Sexual Development, Cell Wall Integrity and Infectious Growth in the Rice Blast Fungus. PloS One.

[78] Chung H (2013). Two conidiation-related Zn (II) 2 Cys 6 transcription factor genes in the rice blast fungus. Fungal Genet. Biol..

[79] Kim HJ (2014). Comparative functional analysis of the velvet gene family reveals unique roles in fungal development and pathogenicity in *Magnaporthe oryzae*. Fungal Genet. Biol..

[80] Samalova, M. *et al*. The β‐1, 3‐glucanosyl transferases (Gels) affect the structure of the rice blast fungal cell wall during appressorium‐mediated plant infection. Cell. Microbiol. 19(3) (2017). 10.1111/cmi.12659 PMC5396357 27568483

[81] Pan Q (1998). Allelism of rice blast resistance genes in two Chinese rice cultivars, and identification of two new resistance genes. Plant Pathol..

[82] Froeliger EH (1996). NUT1, a major nitrogen regulatory gene in *Magnaporthe grisea*, is dispensable for pathogenicity. Mol. Gen. Genet..

[83] Wang ZY (2007). Functional analysis of lipid metabolism in *Magnaporthe grisea* reveals a requirement for peroxisomal fatty acid β-oxidation during appressorium-mediated plant infection. Mol. Plant Microbe Interact.

[84] Terauchi R (2016). Whole genome sequencing approaches to understand *Magnaporthe*-rice interactions. Physiol. Mol. Plant Pathol..

