# Supplementary material for: Large-scale identification of lysine acetylated proteins in vegetative hyphae of the rice blast fungus
Source: Sci Rep. 2017 Nov 10;7:15316. doi: 10.1038/s41598-017-15655-4 (PMC5681509; doi:10.1038/s41598-017-15655-4)
Supplement: Supplementary file 1 — Table S3 [file 41598_2017_15655_MOESM1_ESM.pdf]

Large-scale identification of lysine acetylated proteins in vegetative hyphae of the rice blast fungus

Xiaomei Sun[1*], Zhigang Li[2*], Hang Liu[2], Jun Yang[2], Wenxing Liang[3], You-Liang Peng[2]&Jinguang Huang[3]

Table S1 Gene annotation of acetylated proteins in *Magnaporthe. oryzae*
Table S2 Clusters in protein-protein interaction network
Table S3 List of acetylated proteins potentially involved in vegetative hyphal growth and pathogenicity

Table S3 List of acetylated proteins potentially involved in vegetative hyphal growth and pathogenicity.

Disrupted genes	Mutant ID	Vegetative hyphal growth	Pathogenicity	Annotation
MGG_07200	0421D1	slow	reduced virulence	plasma membrane ATPase
MGG_09901	0100B2	slow	reduced virulence	NAD dependent epimerase/dehydratase
MGG_09991	0236D1	slow	reduced virulence	phosphatidylinositol transfer protein SFH5
MGG_03310	0424D5	slow	reduced virulence	T-complex protein 1 subunit eta
MGG_01062	0082C3	slow	reduced virulence	autophagy-related protein 8
MGG_01061	0082C3	slow	reduced virulence	coproporphyrinogen III oxidase
MGG_00450	0137A2	slow	reduced virulence	phosphoenolpyruvate carboxykinase
MGG_09531	0090D1	slow	reduced virulence	rho-GTPase-activating protein 8
MGG_06204	0249A4	slow	reduced virulence	alpha/beta hydrolase family
MGG_09212	0129C3	slow	reduced virulence	nitroreductase
MGG_04140	0128A4	slow	reduced virulence	mitochondrial NADH dehydrogenase
MGG_02927	0249B2	slow	reduced virulence	putative importin-beta domain-containing protein
MGG_12268	0076D1	slow	wild type	transcription elongation factor spt-5
MGG_08065	0076D1	slow	wild type	WD domain-containing protein
MGG_01712	0135B6	slow	wild type	isocitrate dehydrogenase subunit 2
MGG_08661	0069A4	slow	wild type	acyl-CoA dehydrogenase family member 11
